# [Retracted] Tan IIA inhibits H1299 cell viability through the MDM4‑IAP3 signaling pathway

**DOI:** 10.3892/mmr.2025.13435

**Published:** 2025-01-13

**Authors:** Yukun Zu, Jianning Wang, Wei Ping, Wei Sun

Mol Med Rep 17: 2384–2392, 2018; DOI: 10.3892/mmr.2017.8152

Following the publication of the above paper, it was drawn to the Editors’ attention by a concerned reader that certain of the western blotting data shown in Fig. 1C and D on p. 2386 were strikingly similar to data appearing in different form in a pair of other articles written by different authors at a different research institute that had already been published elsewhere prior to the submission of this paper to *Molecular Medicine Reports*. Moreover, some of the data featured in Fig. 6A and C were strikingly similar, also suggesting that the data in this figure had been misassembled.

In view of the fact that the abovementioned data had already apparently been published previously, the Editor of *Molecular Medicine Reports* has decided that this paper should be retracted from the Journal. The authors were asked for an explanation to account for these concerns, but the Editorial Office did not receive a reply. The Editor apologizes to the readership for any inconvenience caused.

